# New impact factor and Editors of the Journal of Applied Oral Science

**DOI:** 10.1590/1678-77572016ed002

**Published:** 2016

**Authors:** Maria Aparecida A.M. Machado, Carlos F. Santos

**Affiliations:** I Dean of the Bauru School of Dentistry; II Vice-Dean of the Bauru School of Dentistry

On June 14^th^, 2016, the Bauru School of Dentistry (FOB), University of São Paulo (USP) received with huge enthusiasm the great news of the 1.117 impact factor for the Journal of Applied Oral Science (JAOS) in the Journal Citation Reports^®^ (JCR), 2015 Science Edition. Out of the 89 journals indexed in this database in the Dentistry, Oral Surgery & Medicine category, JAOS is now ranked in the 56^th^ place and jumped to the 3^rd^ quartile. Among all the Brazilian journals indexed in JCR, JAOS is the 14^th^ out of 127 journals. It is always worth mentioning that JAOS takes part in the Scientific Electronic Library Online (SciELO), an electronic library covering a selected collection of Brazilian scientific journals, which allows free access to full articles.

Part of this tremendous success is the result of the serious work performed by Dr. Gustavo Pompermaier Garlet and Dr. Karin Hermana Neppelenbroek since they were appointed, in November, 2014, as Editor-in-Chief and Co-Editor-in-Chief, respectively. Another major reason for JAOS’s success is the dedication and professionalism of our employees Valéria Cristina Trindade Ferraz, José Roberto Plácido Amadei, Deborah Schmidt Capella Junqueira, Sônia Cláudia Antonelli Pirola, Neimar Vitor Pavarini, Rubens Kazuo Kato, Allan Rodrigo Dias and Zelma Batista Borges. Finally, since the JAOS is a nonprofit journal, we need to emphasize the financial support provided over the last years by the Rectory of USP and the National Council for Scientific and Technological Development (CNPq).

We also take advantage of this time to announce that Dr. Karin Hermana Neppelenbroek is now officially appointed as the new Editor-in-Chief since Dr. Garlet will face the challenge of serving as an Associate Editor for the Journal of Dental Research, which also made us very proud. Dr. Vanessa Soares Lara will serve the JAOS as the new Co-Editor-in-Chief. We also announce a new panel of Associate Editors, which include colleagues from foreign institutions in order to meet recent criteria established by SciELO. Therefore, we wish the best of luck to the new editors to keep the mission of increasing the international visibility of the JAOS.

Yours sincerely,

Maria Aparecida A.M. Machado Dean of the Bauru School of Dentistry Carlos F. Santos Vice-Dean of the Bauru School of Dentistry

